# SARM1 regulates NAD^+^-linked metabolism and select immune genes in macrophages

**DOI:** 10.1016/j.jbc.2023.105620

**Published:** 2024-01-03

**Authors:** Katharine A. Shanahan, Gavin M. Davis, Ciara G. Doran, Ryoichi Sugisawa, Gavin P. Davey, Andrew G. Bowie

**Affiliations:** School of Biochemistry and Immunology, Trinity Biomedical Sciences Institute, Trinity College Dublin, Dublin, Ireland

**Keywords:** SARM1, NADase, NAD^+^, cADPR, macrophages, metabolism, cytokine induction

## Abstract

Sterile alpha and HEAT/armadillo motif–containing protein (SARM1) was recently described as a NAD^+^-consuming enzyme and has previously been shown to regulate immune responses in macrophages. Neuronal SARM1 is known to contribute to axon degeneration due to its NADase activity. However, how SARM1 affects macrophage metabolism has not been explored. Here, we show that macrophages from *Sarm1*^*−/−*^ mice display elevated NAD^+^ concentrations and lower cyclic ADP-ribose, a known product of SARM1-dependent NAD^+^ catabolism. Further, SARM1-deficient macrophages showed an increase in the reserve capacity of oxidative phosphorylation and glycolysis compared to WT cells. Stimulation of macrophages to a proinflammatory state by lipopolysaccharide (LPS) revealed that SARM1 restricts the ability of macrophages to upregulate glycolysis and limits the expression of the proinflammatory gene interleukin *(Il) 1b*, but boosts expression of anti-inflammatory *Il10*. In contrast, we show macrophages lacking SARM1 induced to an anti-inflammatory state by IL-4 stimulation display increased oxidative phosphorylation and glycolysis, and reduced expression of the anti-inflammatory gene, *Fizz1.* Overall, these data show that SARM1 fine-tunes immune gene transcription in macrophages *via* consumption of NAD^+^ and altered macrophage metabolism.

Macrophages adapt to changing environmental conditions to facilitate effector functions that promote host defense against infection and support tissue homeostasis. These responses require rapid and stimulus-specific changes in gene expression and extensive reprogramming of metabolic activity. For example, during detection of lipopolysaccharide (LPS) from gram-negative bacteria, macrophages rely on aerobic glycolysis as a means of generating energy and upregulate the production of proinflammatory cytokines such as interleukin (IL)-6, tumor necrosis factor and IL-1β. Conversely, in the context of IL-4 stimulation, macrophages exhibit increased oxidative phosphorylation and expression of anti-inflammatory genes such as arginase 1, and resistin-like beta (*Retnlb*, also known as *Fizz1*).

One key contributor to controlling cellular metabolism and transcriptional responses is NAD^+^, which accepts a hydride ion to form its reduced state NADH, an energy transfer intermediate (1). This ability of NAD^+^ to accept hydrides is critical for mediating several metabolic pathways and the regulation and maintenance of NAD^+^ levels in macrophages is expected to be important in fine-tuning cellular responses to a changing environment in the context of immune activation (2).

Intracellular NAD^+^ concentrations depend on a balance between NAD^+^ synthesis and NAD^+^ consumption. Mammalian cells synthesize NAD^+^ *de novo* by the kynurenine pathway or by the Preiss–Handler pathway and recycle it from nicotinamide (Nam) *via* nicotinamide phosphoribosyltransferase in the salvage pathway (3, 4). Depletion of NAD^+^ is mediated by NAD^+^-consuming enzymes, including sirtuins (SIRTs), poly(ADP-ribose) polymerases and the NAD^+^ hydrolases, cluster of differentiation 38 (CD38), and bone-marrow stromal cell antigen 1 (BST1, also known as CD157). Poly(ADP-ribose) polymerases promote DNA repair, transcription, and modulation of chromatin structure, while SIRTs are protein lysine deacetylases that regulate stress responses and energy metabolism (3, 4). CD38 has been implicated in a range of biological functions, including inflammation, ageing, and senescence (4, 5).

Sterile alpha and HEAT/armadillo motif–containing protein (SARM1) was recently described as a NAD^+^-consuming enzyme (6). SARM1 has a tripartite domain structure consisting of an N-terminal armadillo repeat (ARM) region, two central tandem sterile alpha motif domains and a C-terminal toll-IL-1R (TIR) domain (Fig. 1*A*) (7). SARM1 also possesses a hydrophobic and polybasic 27 amino acid N-terminal peptide that is predicted to be a mitochondrial-targeting sequence because of its ability to fold into an α-helix (Fig. 1*A*) (8). TIR domains are normally associated with toll-like receptor signaling, but surprisingly, the TIR domain of SARM1 is actually an NADase (Fig. 1*A*) (6). Indeed, the NAD^+^-hydrolase ability of TIR domains is highly conserved through evolution, with bacteria, plants, and archaea all sharing this catalytic function, indicating that the primordial function of a TIR domain is to metabolize NAD^+^ (9, 10, 11). The glutamate residue at position 642 in human SARM1 (or E682 in mouse SARM1) is required for its catalytic activity and mutation to an alanine is sufficient to abrogate its NADase activity (6). SARM1 cleaves NAD^+^ to generate Nam, ADP ribose and cyclic ADP-ribose (cADPR) (6). The SARM1 TIR domain can also cleave the phosphorylated form of NAD^+^, NADP^+^, and can catalyze the base exchange of Nam for free bases, such as nicotinic acid (12).

**Figure 1:**
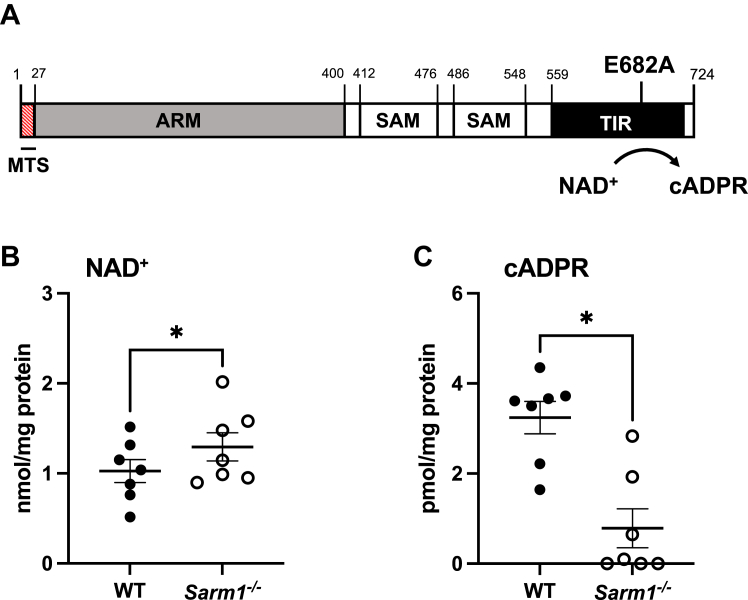
**Macrophages lacking SARM1 display altered cellular NAD**^**+**^**and cADPR levels.***A*, domain organization of murine SARM1. SARM1 contains 724 amino acids and consists of a N-terminal MTS (*red*), an ARM domain (*gray*), two tandem SAM domains (*white*), and a C-terminal TIR domain (*black*). The catalytic residue for the NADase activity of murine SARM1 is a glutamate residue at position 682 (E682) in the TIR domain, which is responsible for cleavage of NAD^+^ to cADPR. *B* and *C*, cellular nucleotides were extracted from WT and *Sarm1*^*−/−*^ BMDM. NAD^+^ levels were determined by HPLC (*B*), and cADPR concentrations were measured by cycling assay (*C*) and values were normalized to protein. Data are from seven independent experiments. Significance tested using two-tailed Wilcoxon matched-pairs signed-rank test; ∗*p* < 0.05. ARM, armadillo repeat; BMDM, bone marrow–derived macrophage; cADPR, cyclic ADP-ribose; MTS, mitochondrial-targeting sequence; SARM1, sterile alpha and HEAT/armadillo motif–containing protein; TIR, toll-interleukin-1R.

SARM1 is most highly expressed in neurons and is also detectable in other cells (13, 14). Within neurons, SARM1 promotes a form of programmed axon degeneration known as Wallerian degeneration (15, 16). Following insult or injury to a neuron, the enzymatic activity of SARM1 is activated by a conformational change, enabling hydrolysis of NAD^+^ (17, 18). Depletion of NAD^+^ leads to loss of ATP, mitochondrial depolarization and metabolic collapse within the axon, which is coupled to axonal degeneration (6).

We previously showed that SARM1 is expressed in murine macrophages, using a mouse expressing epitope-tagged SARM1 endogenously (14). Here, using macrophages from our recently generated SARM1-deficient mice (14), we show a role for SARM1 in maintenance of NAD^+^ and cADPR levels in resting macrophages. We found SARM1 restricts the maximal capacity of oxidative phosphorylation and glycolysis in unstimulated macrophages and by pharmacological inhibition, we show that altering NAD^+^ concentrations, but not blockade of cADPR signaling, influences these parameters. We also find that SARM1 can restrain the upregulation of glycolysis in proinflammatory macrophages, while limiting the expression of *Il1b* and enhancing *Il10* mRNA levels. Further, WT macrophages, compared to *Sarm1*^*−/−*^ cells, stimulated to an anti-inflammatory state display lower levels of oxidative phosphorylation and glycolysis but exhibit increased *Fizz1* expression. Our findings indicate that the enzymatic activity of SARM1 impacts macrophage metabolism, such that SARM1 can fine-tune macrophage immune responses *via* consumption of NAD^+^ and altered metabolism.

## Results

### *Sarm1*^*−/−*^ macrophages display increased cellular NAD^+^ and reduced cADPR levels

We previously demonstrated that SARM1 is expressed in mouse macrophages and showed a role for SARM1 in immune response pathways, assumed to be attributed to TIR domain protein interactions (14, 19). However, whether the NADase activity of SARM1 has any role to play in mouse macrophage function has not yet been explored. In neurons, SARM1 catalyzes the cleavage of NAD^+^, which leads to the generation of Nam and the calcium mobiliser, cADPR (Fig. 1*A*) (6). Therefore, we first examined whether the absence of SARM1 affected cellular NAD^+^ or cADPR concentrations in bone marrow–derived macrophages (BMDMs) from *Sarm1*^*−/−*^ mice that we generated by CRISPR/Cas9-mediated genome editing (14). Metabolites were extracted from WT and *Sarm1*^*−/−*^ BMDM and NAD^+^ was measured by HPLC, while cADPR concentrations were assessed by a fluorescence-based cycling assay. We observed increased cellular levels of NAD^+^ (Fig. 1*B*) and reduced cADPR concentrations (Fig. 1*C*) in unstimulated *Sarm1*^*−/−*^ macrophages compared to WT cells. These results show that removal of SARM1 from unstimulated macrophages is sufficient to alter cellular NAD^+^ and cADPR levels.

### Absence of SARM1 alters macrophage metabolic pathways

**Figure 2:**
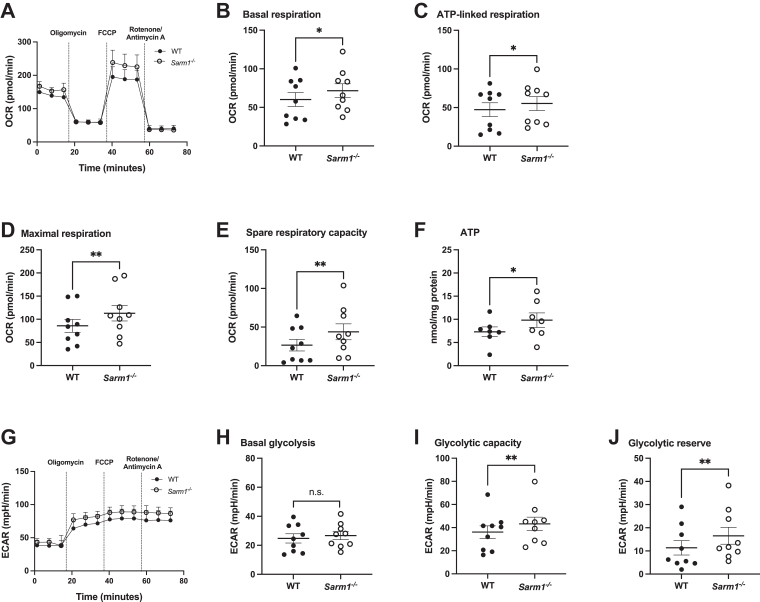
**Removal of SARM1 alters macrophage oxidative phosphorylation and glycolysis.** Real time changes in OCR and ECAR in unstimulated WT and *Sarm1*^*−/−*^ BMDM were measured by Seahorse XF analysis. Representative OCR (*A*) and ECAR (*G*) traces of nine independent experiments and each experiment was performed with six technical replicates. *Dotted lines* indicate injection times of mitochondrial inhibitors, oligomycin (1 μM), FCCP (1 μM), rotenone (1 μM), and antimycin A (2 μM). Basal respiration (*B*), maximal respiration (*C*), ATP-linked respiration (*D*), spare respiratory capacity (*E*), basal glycolysis (*H*), glycolytic capacity (*I*), and glycolytic reserve (*J*) were calculated and displayed as *scattered dot plots*. *B–E* and *H–J*, data are mean ± SEM from nine independent experiments. *F*, cellular nucleotides were extracted from WT and *Sarm1*^*−/−*^ BMDM and ATP levels were determined by HPLC. Data are from seven independent experiments. Significance tested using two-tailed Wilcoxon matched-pairs signed-rank test; ∗*p* < 0.05, ∗∗*p* < 0.01. BMDM, bone marrow–derived macrophage; ECAR, extracellular acidification rate; OCR, oxygen consumption rate; SARM1, sterile alpha and HEAT/armadillo motif–containing protein.

Having shown that macrophage SARM1 contributes to NAD^+^ metabolism, and since NAD^+^ is required to maintain several metabolic pathways (3, 4), we wondered whether SARM1 deletion affected either macrophage oxidative phosphorylation or glycolysis. We analyzed the oxygen consumption rate (OCR), which is indicative of oxidative phosphorylation, in unstimulated BMDM isolated from WT and *Sarm1*^*−/−*^ littermate mice. Maximal respiration and spare respiratory capacity were significantly increased in *Sarm1*^*−/−*^ macrophages compared to WT cells (Fig. 2, *A*, *D* and *E*). Basal respiration and ATP-linked respiration were also enhanced in the absence of SARM1 (Fig. 2, *B* and *C*). In line with increased respiration, we found that cellular ATP levels were heightened in macrophages lacking SARM1 (Fig. 2*F*). We next determined the extracellular acidification rate (ECAR), as an indicator of glycolysis. While basal glycolysis did not differ between WT and *Sarm1*^*−/−*^ BMDM, glycolytic capacity and glycolytic reserve were significantly increased in SARM1-deficient macrophages (Fig. 2, *G–J*). Further evidence that the metabolic differences observed in *Sarm1*^*−/−*^ BMDM were specifically due to the absence of SARM1 came from the fact that mice engineered to express epitope-tagged SARM1 protein in the place of the endogenous gene (14) showed no differences (Fig. S2, *A*–*I*). Together, these data indicate that endogenous SARM1 normally restrains the ability of macrophages to increase either oxidative phosphorylation or glycolysis in response to stress or energy demand.

### SARM1 modulates mitochondrial electron transport complex I activity

**Figure 3:**
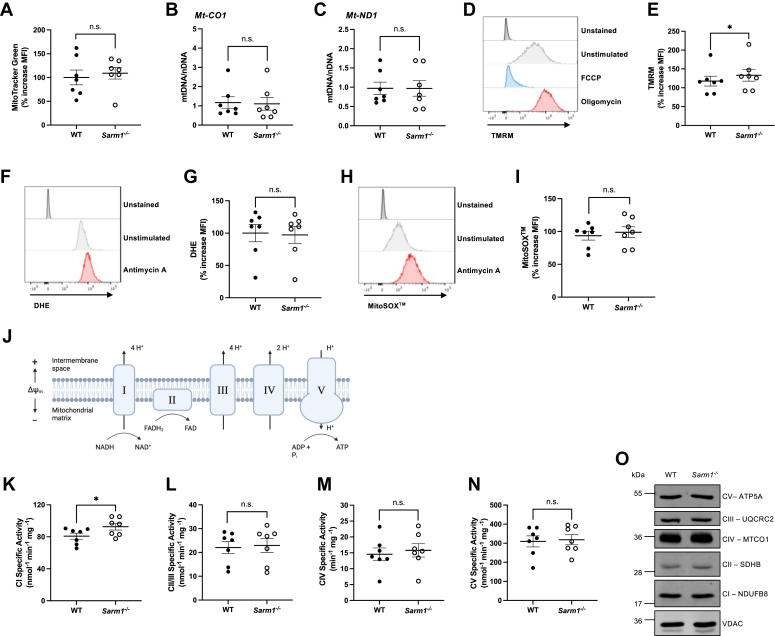
**SARM1-deficient BMDM exhibit increased ΔΨ**_**m**_**and complex I–specific activity, but do not differ in mitochondrial mass or ROS production.***A–C*, mitochondrial mass was assessed in unstimulated WT and *Sarm1*^*−/−*^ BMDM. *A*, cells were stained with 50 nM MitoTracker Green and analyzed using LSRFortessa. Changes in mitochondrial mass were determined by MFI and presented relative to WT cells. *B* and *C*, mitochondrial DNA (*mt-CO1* and *mt-ND1*) was assayed by quantitative RT-PCR and normalized to nuclear DNA. *D* and *E*, ΔΨ_m_ was measured in unstimulated BMDM using 100 nM TMRM and acquired on LSRFortessa. *D*, flow cytometry histogram showing experimental controls for ΔΨ_m_. Oligomycin (1 μM) was used as a positive control for TMRM staining and treatment resulted in a peak shift to the *right*, indicating increased fluorescence. FCCP (50 μM) was used as a negative control and treatment resulted in a peak shift to the *left*, indicating decreased fluorescence. *E*, changes in ΔΨ_m_ between WT and *Sarm1*^*−/−*^ BMDM were determined by MFI and are presented relative to WT cells. *F*, flow cytometry histogram showing experimental controls for cellular ROS. Antimycin A (1 μM) was used as a positive control for DHE staining and treatment resulted in a peak shift to the *right*, indicating increased fluorescence. *G*, unstimulated WT and *Sarm1*^*−/−*^ BMDM were analyzed for cellular ROS using 20 μM DHE and acquired on LSRFortessa. Changes in cellular ROS were determined by MFI and are presented relative to WT cells. *H*, flow cytometry histogram showing experimental controls for mitochondrial ROS. Antimycin A (1 μM) was used as a positive control for MitoSOX staining and treatment resulted in a peak shift to the *right*, indicating increased fluorescence. *I*, unstimulated WT and *Sarm1*^*−/−*^ BMDM were analyzed for changes in mitochondrial ROS using 5 μM MitoSOX and acquired on LSRFortessa. Changes in mitochondrial ROS were determined by MFI and are presented relative to WT cells. *J*, diagram of ETC. *K–O*, mitochondria were isolated from WT and *Sarm1*^*−/−*^ BMDM and protein was quantified by Bradford assay. Ten micrograms of mitochondrial protein was added to each assay. Complex I (*K*), complex II/III (*L*), complex IV (*M*), and complex V (*N*) activities were determined spectrophotometrically and presented as nmol per mg of mitochondrial protein. *O*, representative immunoblot of three biological replicates. Ten micrograms of mitochondrial protein was immunoblotted for components of the ETC: NDUFB8 (complex I subunit), SDHB (complex II subunit), UQCRC2 (complex III subunit), MTCO1 (complex IV subunit), and ATP5A (complex V) and VDAC as the loading control. *A–C*, *E*, *G*, *I*, *K–N*, *scattered dot plots* show mean ± SEM from seven independent experiments (n = 7) and each experiment was performed with three technical replicates. Significance tested using two-tailed Wilcoxon matched-pairs signed-rank test; ∗*p* < 0.05, n.s., no significant difference. BMDM, bone marrow–derived macrophage; ETC, electron transport chain; MF1, median fluorescence intensity; SARM1, sterile alpha and HEAT/armadillo motif–containing protein; TMRM, tetramethylrhodamine methyl ester.

Enhanced NAD^+^ levels have been linked to increased mitochondrial function (1, 3). Since NAD^+^ concentrations and mitochondrial respiration were elevated in *Sarm1*^*−/−*^ cells, we examined other mitochondrial parameters in resting WT and *Sarm1*^*−/−*^ littermate BMDM. First, we analyzed mitochondrial mass by MitoTracker Green staining and the ratio of mitochondrial and nuclear DNA. There were no significant differences in mitochondrial mass between WT and *Sarm1*^*−/−*^ macrophages, indicating that the observed increase in mitochondrial respiration in SARM1-deficient cells was not because of alterations in mitochondrial mass (Fig. 3, *A–C*). Next, we assessed mitochondrial membrane potential (ΔΨ_m_) using tetramethylrhodamine methyl ester (TMRM) staining. Oligomycin, which inhibits ATP synthase, was used as a positive control as it causes proton accumulation which results in an increase in ΔΨ_m_. The mitochondrial uncoupler, carbonyl cyanide *p*-trifluoromethoxyphenylhydrazone (FCCP), was used as a negative control because it depolarizes mitochondrial membranes (Fig. 3*D*). ΔΨ_m_ was significantly increased in *Sarm1*^*−/−*^ cells compared to their WT counterparts (Fig. 3*E*), suggesting that mitochondrial activity is enhanced when SARM1 is removed from macrophages. We then measured cellular reactive oxygen species (ROS) levels using dihydroethidium (DHE) staining and mitochondrial ROS levels by MitoSOX staining. Antimycin A, a potent complex III inhibitor, generates superoxide and was used as a positive control for both cellular and mitochondrial ROS (Fig. 3, *F* and *H*). We observed no significant differences in cellular or mitochondrial ROS production between WT and *Sarm1*^*−/−*^ BMDM (Fig. 3, *G* and *I*). Since elevated ROS can be associated with mitochondrial dysfunction, the normal ROS levels in SARM1-deficient cells suggest they have healthy mitochondria. To test why ΔΨ_m_ was increased in macrophages lacking SARM1, we investigated the function of the electron transport chain (ETC) and ATP synthase using enzymatic assays that determine the specific ETC complex activities in pure mitochondria isolated from WT and *Sarm1*^*−/−*^ BMDM. SARM1-deficient mitochondria showed a selective increase only in complex I activity (NADH dehydrogenase), and not complex II/III, complex IV, or complex V (ATP synthase) (Fig. 3, *K–N*). Immunoblot analysis of the complex I subunit, NDUFB8, confirmed that enhanced complex I activity in *Sarm1*^*−/−*^ mitochondria was not due to increased protein expression (Fig. 3*O*). We also found no differences in expression of subunits of complex II (SDHB), complex III (UQCRC2), complex IV (MTCO1), or complex V (ATP5A) between WT and *Sarm1*^*−/−*^ mitochondria (Fig. 3*O*). Taken together, these findings indicate that removal of SARM1 from macrophages increases complex I activity, ΔΨ_m_, and mitochondrial respiration.

### Depletion of cellular NAD^+^ reduces maximal respiration and glycolysis in macrophages

We then asked if alterations in metabolic readouts in *Sarm1*^*−/−*^ BMDM could be due to either elevated NAD^+^ or reduced cADPR levels. To examine the latter possibility, we treated WT macrophages with the cell permeable antagonist of cADPR, 8-Bromo-cADPR (8-Br-cADPR) and examined oxidative phosphorylation and glycolysis following treatment. Pharmacological blockade of cADPR signaling by 8-Br-cADPR did not reduce basal or maximal respiration (Fig. S4, *A–C*). Glycolysis and glycolytic capacity were also unaffected after 8-Br-cADPR treatment (Fig. S4, *D–F*). Thus, lower cADPR concentrations in *Sarm1*^*−/−*^ cells are unlikely to explain the increase in reserve capacity of oxidative phosphorylation and glycolysis that we observed.

**Figure 4:**
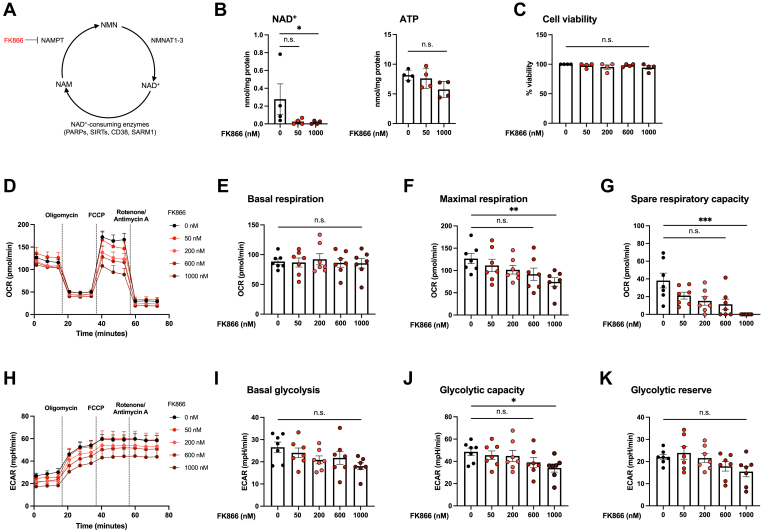
**Depletion of cellular NAD**^**+**^**reduces maximal respirationand glycolysis in macrophages.***A*, schematic of the NAD^+^ salvage pathway, indicating the point at which FK866 inhibits. *B*, BMDM were treated with 50 nM or 1000 nM FK866 for 18 h, followed by nucleotide extraction. NAD^+^ and ATP concentrations determined by HPLC analysis and values were normalized to protein. *C*, WT BMDM were treated with indicated concentrations of FK866 for 18 h and cell viability was measured by viability staining using Zombie Aqua. *D–K*, WT BMDM were treated with indicated concentrations of FK866 for 18 h and real-time changes in OCR and ECAR were measured by Seahorse XF analysis. Representative OCR (*D*) and ECAR (*H*) trace of seven independent experiments and each experiment was performed with five technical replicates. *Dotted lines* indicate injection times of mitochondrial inhibitors, oligomycin (1 μM), FCCP (1 μM), rotenone (1 μM), and antimycin A (2 μM). Basal respiration (*E*), maximal respiration (*F*), spare respiratory capacity (*G*), basal glycolysis (*I*), glycolytic capacity (*J*), and glycolytic reserve (*K*) were calculated and displayed as *bar charts*. *B* and *C*, data are mean ± SEM from four independent experiments and each experiment was performed with three technical replicates. *E−G* and *I–K*, data are mean ± SEM from seven independent experiments and each experiment was performed with five technical replicates. Significance tested using Kruskal–Wallis test; ∗*p* < 0.05, ∗∗*p* < 0.01, and ∗∗∗*p* < 0.001, n.s., no significant difference. BMDM, bone marrow–derived macrophage; ECAR, extracellular acidification rate; OCR, oxygen consumption rate.

We next considered whether the metabolic differences between WT and *Sarm1*^*−/−*^ macrophages could be attributed to altered levels of cellular NAD^+^. To examine this hypothesis, we used the specific inhibitor of nicotinamide phosphoribosyltransferase, FK866, which has been shown to efficiently decrease cellular NAD^+^ (Fig. 4*A*) (20). We selected a range of concentrations of FK866 from 50 nM to 1 μM and confirmed that treatment of WT macrophages with either the highest or lowest concentration of FK866 reduced intracellular NAD^+^ (Fig. 4*B*). Cell viability was not affected in FK866-treated macrophages, even at the highest concentration chosen, indicating that changes observed in subsequent metabolic experiments would not be due to reduced cell viability (Fig. 4*C*). We then examined macrophage oxidative phosphorylation and glycolysis following NAD^+^ depletion. FK866 treatment reduced maximal respiration and spare respiratory capacity, while basal and ATP-linked respiration were unaffected. Macrophage glycolytic capacity was reduced in a concentration-dependent manner. These data provide evidence that differences in the reserve capacity of oxidative phosphorylation and glycolysis between unstimulated WT and *Sarm1*^*−/−*^ BMDM are likely due to altered NAD^+^ levels and not cADPR concentrations.

### SARM1 influences metabolic reprogramming and expression of select genes in proinflammatory and anti-inflammatory macrophages

Thus far, our data has shown that (1) SARM1 reduces cellular NAD^+^ levels in unstimulated macrophages (2), SARM1 limits the maximal capacity of oxidative phosphorylation and glycolysis in macrophages and (3) lowering macrophage NAD^+^ levels reduces the reserve capacity of oxidative phosphorylation and glycolysis. NAD^+^ is necessary to support macrophage metabolic reprogramming of proinflammatory and anti-inflammatory macrophages and influences the expression of several proinflammatory and anti-inflammatory genes (21, 22). Thus, we wondered if SARM1-deficient macrophages would display altered metabolism or gene expression following stimulation with either LPS or IL-4, which generates a proinflammatory or anti-inflammatory state in macrophages, respectively.

**Figure 5:**
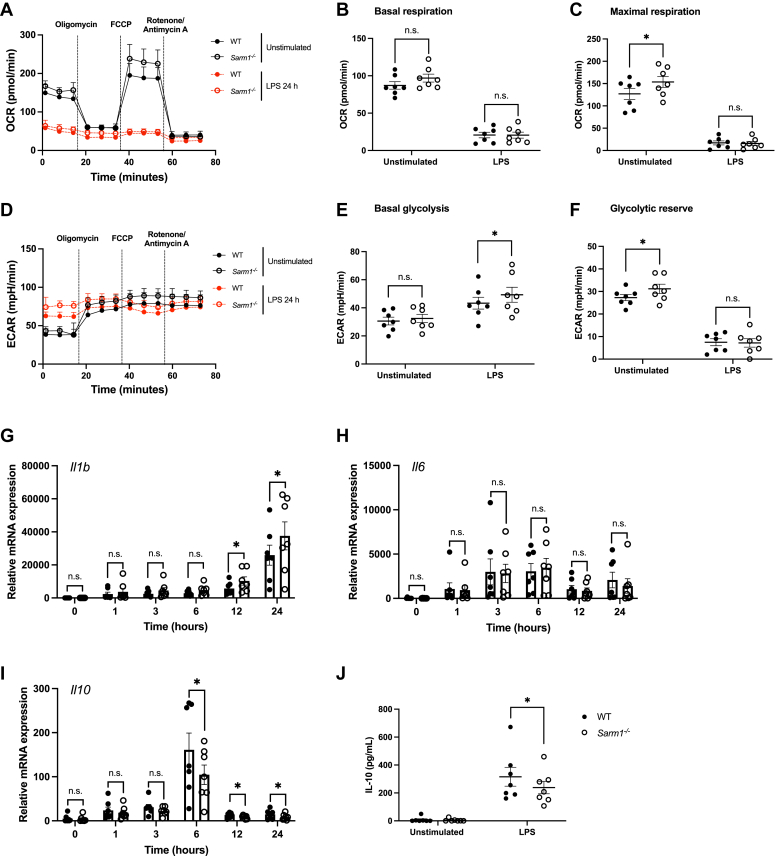
**SARM1 inhibits the upregulation of glycolysis in proinflammatory macrophages and alters the expression of *Il1b* and *Il10*.***A–F*, WT and *Sarm1*^*−/−*^ BMDM were treated with 100 ng/ml LPS for 24 h and real-time changes in OCR and ECAR were measured by Seahorse XF analysis. Representative OCR (*A*) and ECAR (*D*) trace of seven independent experiments and each experiment was performed with six technical replicates. *Dotted lines* indicate injection times of mitochondrial inhibitors, oligomycin (1 μM), FCCP (1 μM), rotenone (1 μM), and antimycin A (2 μM). Basal respiration (*B*), maximal respiration (*C*), basal glycolysis (*E*), and glycolytic reserve (*F*) were calculated and displayed as *scattered dot plots*. Data are mean ± SEM from seven independent experiments and each experiment was performed with six technical replicates. *G–I*, WT and *Sarm1*^*−/−*^ BMDM were treated with 100 ng/ml LPS for the indicated times. Expression of *Il1b* (*G*), *Il6* (*H*), and *Il10* (*I*) mRNA were assayed by quantitative RT-PCR and normalized to the housekeeping gene β-actin. *J*, WT and *Sarm1*^*−/−*^ BMDM were stimulated with 100 ng/ml LPS for 24 h. Supernatants were assayed for IL-10 protein. Data are mean ± SEM from seven independent experiments and each experiment was performed with three technical replicates. Significance tested using two-tailed Wilcoxon matched-pairs signed-rank test; ∗*p* < 0.05, n.s., no significant difference. BMDM, bone marrow–derived macrophage; ECAR, extracellular acidification rate; IL, interleukin; LPS, lipopolysaccharide; OCR, oxygen consumption rate; SARM1, sterile alpha and HEAT/armadillo motif-containing protein.

To test this, we treated WT and *Sarm1*^*−/−*^ BMDM with LPS for 24 h and examined OCR and ECAR. Oxidative phosphorylation was downregulated in both LPS-treated WT and *Sarm1*^*−/−*^ BMDM (Fig. 5, *A–C*). Glycolysis was significantly boosted in *Sarm1*^*−/−*^ BMDM following LPS treatment compared to WT macrophages (Fig. 5, *D* and *E*). We then wondered whether expression of LPS-induced genes was affected in the absence of SARM1. To check this, we performed a 24 h LPS time course on WT and *Sarm1*^*−/−*^ BMDM. We found that *Il1b* transcription was significantly increased in SARM1-deficient macrophages relative to WT cells following LPS treatment at 12 and 24 h, but not at earlier timepoints (Fig. 5*G*). Not all proinflammatory genes were affected in *Sarm1*^*−/−*^ cells since there was no difference in *Il6* expression compared to WT counterparts (Fig. 5*H*). Conversely, induction of the anti-inflammatory gene, *Il10*, was significantly reduced in LPS-stimulated *Sarm1*^*−/−*^ macrophages relative to WT at 6 h, 12 h, and 24 h LPS treatment, but not at earlier time points (Fig. 5*I*). LPS-induced IL-10 secretion was also limited at 24 h in macrophages lacking SARM1 (Fig. 5*J*). These findings suggest that SARM1 restrains the upregulation of glycolysis in proinflammatory macrophages, as well as reducing the expression of *Il1b* and enhancing *Il10* mRNA levels.

**Figure 6:**
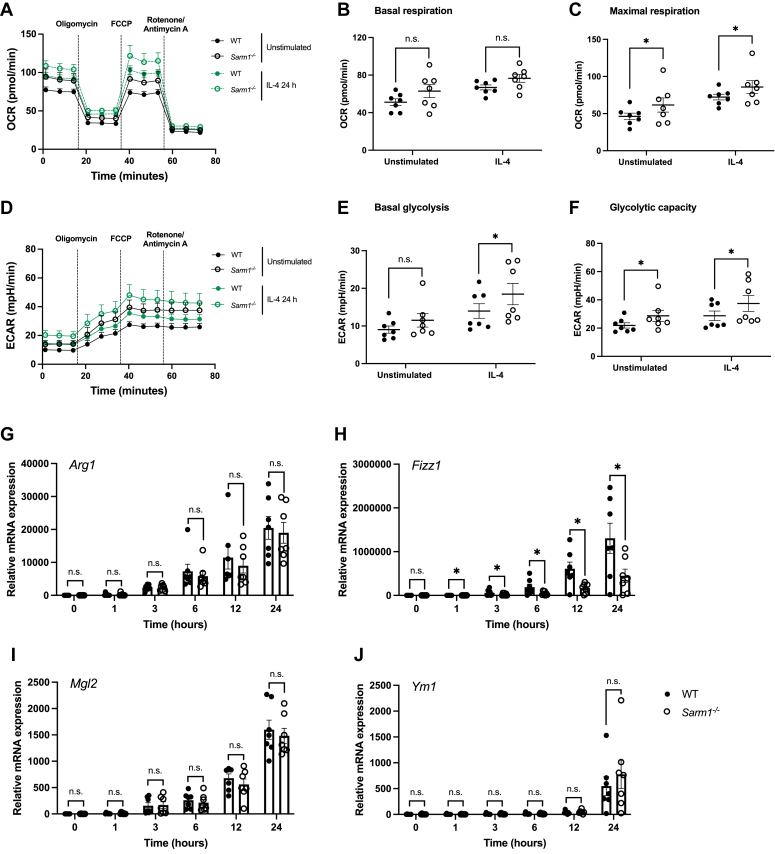
**Anti-inflammatory macrophages lacking SARM1 display increased oxidative phosphorylation and glycolysis, but reduced *Fizz1* expression.***A–F*, WT and *Sarm1*^*−/−*^ BMDM were treated with 20 ng/ml IL-4 for 24 h and real-time changes in OCR and ECAR were measured by Seahorse XF analysis. Representative OCR (*A*) and ECAR (*D*) trace of seven independent experiments and each experiment was performed with six technical replicates. *Dotted lines* indicate injection times of mitochondrial inhibitors, oligomycin (1 μM), FCCP (1 μM), rotenone (1 μM), and antimycin A (2 μM). Basal respiration (*B*), maximal respiration (*C*), basal glycolysis (*E*), and glycolytic capacity (*F*) were calculated and displayed as *scattered dot plots*. Data are mean ± SEM from seven independent experiments and each experiment was performed with six technical replicates. *G–J*, WT and *Sarm1*^*−/−*^ BMDM were treated with 20 ng/ml IL-4 for the indicated times. Expression of *Arg1* (*G*), *Fizz1* (*H*), *Mgl2* (*I*), and *Ym1* (*J*) mRNA were assayed by quantitative RT-PCR and normalized to the housekeeping gene β-actin. Significance tested using two-tailed Wilcoxon matched-pairs signed-rank test; ∗*p* < 0.05, n.s., no significant difference. BMDM, bone marrow–derived macrophage; ECAR, extracellular acidification rate; IL, interleukin; OCR, oxygen consumption rate; SARM1, sterile alpha and HEAT/armadillo motif-containing protein.

We next examined whether SARM1 restricted macrophage metabolic reprogramming of anti-inflammatory macrophages. We stimulated WT and *Sarm1*^*−/−*^ BMDM with IL-4 for 24 h and measured OCR and ECAR. Oxidative phosphorylation was enhanced in SARM1-deficient BMDM compared to WT cells, with maximal respiration remaining significantly elevated (Fig. 6, *A–C*). Glycolysis and glycolytic capacity were also significantly increased in *Sarm1*^*−/−*^ BMDM compared to WT macrophages following IL-4 stimulation (Fig. 6, *D–F*). We next carried out a 24 h IL-4 time course on macrophages derived from WT and *Sarm1*^*−/−*^ littermate mice. Expression of *Fizz1*, one of the most abundantly IL-4–induced transcripts in macrophages, was significantly reduced in BMDM lacking SARM1 (Fig. 6*H*). There were no significant differences in mRNA levels of IL-4 stimulated arginase 1, galactose N-acetyl-galactosamine-specific lectin 2 (*Mgl2*), or chitinase 3-like 3 (*Chil3*, also known as *Ym1*), indicating that there is not a global impairment in induction of anti-inflammatory genes in macrophages from *Sarm1*^*−/−*^ mice (Fig. 6, *G*, *I* and *J*). Thus, removal of SARM1 allows for enhanced oxidative phosphorylation and glycolysis in anti-inflammatory macrophages and limits the expression of the anti-inflammatory gene, *Fizz1*.

## Discussion

To date, studies of SARM1 have focused on a role for its NADase in neurons, but a role for this enzymatic activity in macrophages has not yet been described. Here, we show that in macrophages (1) SARM1 deletion elevates baseline NAD^+^ concentrations and reduces cADPR levels; (2) SARM1 reduces the reserve capacity of oxidative phosphorylation and glycolysis, and can limit complex I specific activity, ΔΨ_m_ and ATP levels; (3) SARM1 limits the upregulation of glycolysis in proinflammatory macrophages, and this is accompanied by reduced *Il1b* expression and enhanced levels of *Il10*, suggesting SARM1 restrains LPS-stimulated inflammatory responses; (4) anti-inflammatory macrophages lacking SARM1 display enhanced oxidative phosphorylation and glycolysis and diminished expression of the anti-inflammatory gene, *Fizz1* (Fig. S5).

It is assumed that SARM1-dependent NAD^+^ depletion in cells requires activation of SARM1’s enzyme activity, triggered by an imbalance of the NMN-to-NAD^+^ ratio (18, 23). However, SARM1 may display basal enzyme activity in certain contexts. A previous study reported that NAD^+^ levels were modestly increased in brain tissue isolated from *Sarm1*^*−/−*^ mice compared to WT mice, and cADPR concentrations were significantly decreased in uninjured *Sarm1*^*−/−*^ primary neurons, brain tissue, and sciatic nerve (24). SARM1 deletion also elevates NAD^+^ pools in cardiac tissue from female, and to a lesser extent, in male knock-out mice (25). In our study, here, we found that macrophages lacking SARM1 displayed increased cellular levels of NAD^+^ and decreased concentrations of cADPR in the absence of stimulation, suggesting that SARM1 has a low level of basal NADase activity in macrophages. Thus, SARM1 regulates macrophage NAD^+^ under physiological conditions, and its deletion leads to increased cellular NAD^+^ concentrations.

Elevated NAD^+^ levels are associated with increased mitochondrial health and function. In ageing and metabolic diseases, decreased synthesis and increased consumption of NAD^+^ leads to depletion of NAD^+^ stores and decline in mitochondrial function. Ageing macrophages have reduced expression of the NAD^+^-synthesizing enzyme, quinolate phosphoribosyltransferase, which is linked to lower basal respiration and complex I activity (26). In aged mice, expression and activity of the NAD^+^-consuming enzyme, CD38, are increased and *Cd38*^*−/−*^ mice have elevated mitochondrial NAD^+^, ΔΨ_m_, and OCR without affecting the number of mitochondria (27). Similarly, we observe that removal of SARM1 from macrophages increases NAD^+^ levels, complex I activity, ΔΨ_m_ and mitochondrial respiration, but does not affect mitochondrial mass. This is likely due to increased NAD^+^ availability and increased NADH respiration since there was no difference in complex II/III activity. The increase in mitochondrial activity we observed, without increased ROS production, suggests that SARM1 deletion exerts positive control over mitochondrial function and bioenergetics. Like CD38, SARM1 expression or activity may increase in ageing or metabolic disease and SARM1 could also be involved in pathologies related to NAD^+^ decline.

NAD^+^ metabolism influences macrophage metabolic reprogramming, which drives their proinflammatory or anti-inflammatory state during macrophage activation (21, 22). Lowering NAD^+^ levels by inhibition of the NAD^+^ salvage pathway restricts metabolic reprogramming of proinflammatory and anti-inflammatory macrophages, and this can be restored by supplementation with the NAD^+^ precursor, NMN (21, 22). Our data show that SARM1 limits basal NAD^+^ concentrations and perhaps this modulation of NAD^+^ levels by SARM1 is another mechanism by which macrophages regulate their metabolic reprogramming. Consistent with this notion, glycolysis and oxidative phosphorylation are enhanced in SARM1-deficient macrophages following stimulation. Interestingly, SARM1 expression is downregulated following stimulation with either LPS or IL-4 (data not shown), and this may be one of the ways macrophages increase glycolysis and oxidative phosphorylation during macrophage activation.

Modulation of the NAD^+^ salvage pathway can also influence the abundance of several proinflammatory and anti-inflammatory genes. We found that SARM1 regulates expression of select genes, namely proinflammatory *Il1b* and anti-inflammatory *Il10* and *Fizz1* (this work and (14)). How SARM1-mediated altered metabolism regulates transcription of these genes in not fully clear. One possibility is that SARM1 affects the expression of, or NAD^+^ supply to, other NAD^+^-dependent enzymes, such as SIRTs, which play important roles in epigenetic modifications. Indeed, mRNA levels of several SIRTs are reduced in cardiac tissue isolated from *Sarm1*^*−/−*^ mice (25) and inhibition of SIRT1/2 can enhance *Il1b* as well as reduce IL-10 production and *Fizz1* expression (22, 28). Alternatively, accumulation of tricarboxylic acid cycle intermediates influence gene expression and SARM1 may modulate some of these, like succinate which increases LPS-induced *I11b* and suppresses *Il10* (29).

Our data point toward SARM1 as a regulatory mechanism in macrophages to limit the proinflammatory response, which is likely due to control of cellular NAD^+^ concentrations. However, another mechanism by which SARM1 could regulate cellular processes is by the generation of cADPR or by minor glycocyclic ADP-ribose products (30). In the case of CD38, cADPR has been shown to control neutrophil chemotaxis following bacterial infection (31) and CD38-induced cADPR generation augmented the proinflammatory response in response to respiratory syncytial virus (32). Thus, SARM1’s regulation of cADPR in the context of macrophage activation merits exploration.

Overall, our study demonstrates a role for SARM1 in regulation of macrophage NAD^+^ metabolism, which influences metabolic reprogramming and the expression of select proinflammatory and anti-inflammatory genes. These findings reveal a previously uncharacterized role for the NADase activity of SARM1 in macrophages.

## Experimental procedures

### Mice

C57BL/6 mice lacking *Sarm1* generated by CRISPR/Cas9 genome editing were described previously (14) and are referred to as *Sarm1*^*−/−*^ throughout the text. Mice were housed in individually ventilated cages (Tecniplast) in a specific pathogen-free facility on a 12 h light/dark cycle with access to food and water *ad libitum* at the Trinity Biomedical Sciences Institute, Trinity College Dublin. For experiments, BMDM from CRISPR/Cas9 *Sarm1*^*−/−*^ mice were compared to WT littermates from heterozygous breeding. Experiments for Figures 1*C* and 6, *A–F* were performed using BMDM from mice from both heterozygous breeding pairs and homozygous breeding pairs. Male and female mice were used for experiments and all animals used were 8 to 10 weeks old. Procedures were conducted under licenses from the Irish Health Products Regulatory Authority (Reference: AE19136/P083) and approved by Trinity College Dublin’s Animal Research Ethics Committee.

### Generation and culture of BMDMs

To generate primary BMDM, bone marrow was flushed from femurs and tibiae of mice using a 27-gauge needle and a syringe filled with Dulbecco's modified Eagle's medium (DMEM) with GlutaMAX supplement (Gibco). Clumps were gently disaggregated using a 19-gauge needle and syringe. The cell suspension was centrifuged at 250*g* for 5 min at room temperature and incubated with red blood cell lysis buffer (Miltenyi Biotec). Cells were counted and cultured in DMEM with GlutaMAX supplement, 10% (v/v) fetal calf serum (Gibco), 1% (v/v) penicillin-streptomycin (Sigma-Aldrich), 100 μg/ml normocin (InvivoGen), and supplemented with 20% (v/v) L929 supernatant as a source of macrophage colony-stimulating factor and incubated at 37 °C with 5% CO_2_. On day 6 or day 7, BMDM were removed from flasks using a sterile cell scraper, counted, and were seeded for experiments in complete DMEM-containing 20% (v/v) L929.

### Cell treatments

FK866 and 8-Br-cADPR were used at the indicated concentrations. LPS serotype EH(100)Ra (ENZO) was used at 100 ng/ml and IL-4 (PeproTech) was used at 20 ng/ml.

### Nucleotide extraction

Cells (5 × 10^6^ BMDM for NAD^+^ and ATP measurements, or 40 × 10^6^ BMDM for cADPR detection) were seeded and allowed to adhere overnight. Cells were scraped in 1× PBS, washed once in 1× PBS, and resuspended in 500 μl of 1× PBS. Twenty microliters of the cell suspension was taken for a protein sample. Cells were pelleted, resuspended in 555 μl of 0.5 M perchloric acid, and incubated on ice for 5 min, vortexing briefly every minute. Samples were spun at 21,130*g* for 15 min at 4 °C. Five hundred microliters of the supernatant was removed and neutralized using 2.5 M potassium hydroxide and incubated on ice for 5 min, vortexing briefly every minute. Samples were spun at 21,130*g* for 15 min at 4 °C to remove salt formed during neutralization and were either analyzed directly or stored at −80 °C until analysis.

### HPLC analysis

NAD^+^ and ATP concentrations were measured by HPLC analysis on a C18 Gemini 3 μm column (Phenomenex), using an isocratic method adapted from Ingebretson *et al.*, 1982 (33). The mobile phase consisted of 220 mM potassium phosphate, 0.3 mM tetrabutylammonium, and 1% methanol, pH 6.9. The method was run for 30 min at a flow rate of 1 ml/min at 30 °C. Chromatograms were analyzed using Empower Pro Software (www.waters.com). Metabolite level was normalized to protein concentration measured by Pierce bicinchoninic acid (BCA) Protein Assay Kit (Thermo Fisher Scientific).

NAD^+^ in the text refers to total cellular NAD^+^ and NADH, since NADH is oxidized to NAD^+^ during perchloric acid precipitation.

### Cycling assay for cADPR measurement

cADPR concentrations were measured by a fluorescence-based cycling assay described previously (34). Following nucleotide extraction, the pH was adjusted to pH 8 by adding 400 μl of 10 mM sodium phosphate, pH 8. To remove contaminating nucleotides, 0.2 U/ml phosphodiesterase (Sigma-Aldrich) and 0.5 M MgCl_2_ was added to each sample and samples were incubated overnight at 37 °C with shaking. Phosphodiesterase was removed by filtration with 0.5 ml Amicon Ultra (3 K) and samples were recovered in the filtrate after centrifugation at 3000*g* for 30 min at 4 °C. Prior to preparing the cycling reagent, NAD^+^ was removed from alcohol dehydrogenase (ADH) (Sigma-Aldrich) and diaphorase (Sigma-Aldrich) by charcoal treatment.

Reactions were conducted in 96-well plates. To 100 μl of sample, 50 μl of reagent containing 0.3 μg/ml ADP-ribosyl cyclase (Sigma-Aldrich), 30 nM Nam (Sigma-Aldrich), and 100 mM sodium phosphate, pH 8 was added and incubated for 15 min at 25 °C. As a control for background fluorescence, each set of samples had one well that contained 100 μl of sample and 50 μl of reagent containing 30 mM Nam and 100 mM sodium phosphate, pH 8. Then, 100 μl of cycling reagent containing 100 μg/ml ADH, 2% ethanol, 20 μM resazurin (TCI), 10 μg/ml diaphorase, 10 μM flavin mononucleotide (Sigma-Aldrich), 10 μM Nam, 0.1 mg/ml bovine serum albumin (BSA), and 100 mM sodium phosphate, pH 8 was added to each well. The cycling reaction was allowed to proceed for 1 to 12 h and the increase in fluorescence was measured every hour using SpectraMax fluorescence plate reader and SoftMax Pro 4.8 software (www.moleculardevices.com), with excitation at 544 nm and emission at 590 nm. Metabolite level was normalized to protein concentration measured by Pierce BCA Protein Assay Kit (Thermo Fisher Scientific).

### Metabolic flux analysis

Real-time OCR and glycolytic flux (ECAR) of BMDM were measured using Seahorse XF-96 Extracellular Flux Analyser (Agilent). Cells (5 × 10^5^ per ml) were seeded per well of a Seahorse XF-96 cell culture plate (Agilent) in complete DMEM. For unstimulated BMDM, cells were left to adhere overnight. For treated BMDM, cells were allowed to adhere for 6 h prior to treatment with FK866 or 8-Br-cADPR for 18 h or stimulation with LPS or IL-4 for 24 h.

The day before analysis, a utility plate containing the injector ports and probes was filled with calibration fluid, pH 7.4 (Agilent) and was placed in a non-CO_2_ incubator at 37 °C overnight.

The following day, complete DMEM was removed from cells and replaced with 180 μl of XF assay media (pH 7.4) supplemented with 1 mM pyruvate, 2 mM L-glutamine, and 0.01 M glucose. Seahorse XF-96 cell culture plate was then placed in a non-CO_2_ incubator 1 h prior to running the assay to remove oxygen. Oligomycin (1 μM), FCCP (1 μM), rotenone (1 μM), and antimycin A (2 μM) were added to the appropriate ports of the utility plate for a standard Seahorse XF Cell Mito Stress Test. Sequential measurements of OCR and ECAR following the addition of the inhibitors enabled calculation of basal mitochondrial respiration, ATP-linked respiration, maximal mitochondrial respiration, spare respiratory capacity, basal glycolysis, glycolytic capacity, and glycolytic reserve (Fig. S1). Results were collected with Wave software version 2.4.3.3 (www.agilent.com).

For experiments comparing WT and *Sarm1*^*−/−*^ BMDM, OCR and ECAR readings were normalized using absorbance readings obtained from Pierce BCA Protein Assay Kit (Thermo Fisher Scientific). Briefly, media was aspirated from the wells, 50 μl of radioimmunoprecipitation assay buffer was added to each well and the plate was frozen at −20 °C for 30 min or overnight. The plate was defrosted and centrifuged at 300*g* for 5 min. One hundred microliteres of Pierce BCA reagent was added to each well and the plate was incubated at 37 °C for 30 min. Absorbance was read at 562 nm on the VersaMax Microplate Reader using the SoftMax Pro 7.03 software (www.moleculardevices.com). For scattered dot plots in Figures 2, 5 and 6, each data point is an experimental and a biological replicate and is an average of six technical replicates. For statistical analysis, the averaged experimental and biological pairs were compared.

### Flow cytometry

BMDM were plated at 5 × 10^5^ cells/ml and allowed to adhere overnight. The following day, media was removed, and cells were washed once in 1× PBS. Cells were scraped into 500 μl of 1× PBS, transferred to round-bottom polypropylene test tubes (Thermo Fisher Scientific) and pelleted at 400*g*. Cells were stained with 100 nM MitoTracker Green (for mitochondrial mass), 100 nM TMRM (for ΔΨ_m_), 20 μM DHE (for cellular ROS), or 5 μM MitoSOX (for mitochondrial ROS). Samples were incubated at 37 °C for 30 min and protected from light. For cell viability, cells were stained with Zombie Aqua at room temperature for 15 min. Following staining, cells were washed once, resuspended in 300 μl 1× PBS and acquired on LSRFortessa using (488) 530/30 for MitoTracker Green, (561) 610/20 for TMRM, (561) 582/15 for DHE and MitoSOX and (405) 525/50 nm for Zombie Aqua. As a control for TMRM staining, oligomycin (1 μM) or FCCP (50 μM) were added directly to cells prior to acquisition. As a control for DHE and MitoSOX staining, antimycin A (1 μM) was added directly to cells prior to acquisition. For Zombie Aqua, 100% ethanol was added directly to cells as a positive control for live/dead staining. Data were analyzed using FlowJo v10 Software (www.flowjo.com). A flow gating strategy is provided in Fig. S3*A* for untreated cells (Fig. 3, *A*, *E*, *G* and *I*) and Fig. S3*B* for cell viability following FK866 treatment (Fig. 4*C*).

### Mitochondrial/nuclear DNA

Total DNA was extracted from 5 × 10^6^ cells using DNeasy Blood & Tissue Kit (QIAGEN) according to the manufacturers’ instructions. The concentration of DNA was determined using the Nanodrop 2000 (Thermo Fisher Scientific). Mitochondrial DNA was assessed by real-time PCR analysis to measure the abundance of specific mitochondrial encoded genes relative to a nuclear encoded gene (sequences are listed in Table S1).

### Mitochondrial isolation

All following steps were carried out on ice or at 4 °C. BMDMs (40 × 10^6^) were harvested, washed once in 1× PBS, and resuspended in 1 ml buffer containing 320 nM sucrose, 10 mM Tris, 1 mM EDTA, and supplemented with protease inhibitors (10 μl/ml aprotinin, 1 mM sodium orthovanadate, and 1 mM PMSF). Samples were transferred to a glass Dounce homogenizer with a glass pestle and homogenized with 30 strokes. Cell lysates were centrifuged at 700*g* for 10 min, and the resulting supernatants were removed and centrifuged at 12,000*g* for 15 min to isolate the mitochondrial fraction. Mitochondria were freeze-thawed thrice using liquid nitrogen prior to analysis.

### Respiratory chain complex activity assays

Complex I activity assay was based on a protocol described by Spinazzi *et al.* (35). The assay measures the oxidation of NADH to NAD^+^ with concomitant decrease in absorbance at 340 nm at 37 °C. In a 1 ml cuvette, the reaction mixture contained assay buffer, 25 mM phosphate, 0.2 mM NADH, 2.5 mg/ml fatty acid–free BSA, 1 mM KCN, and 10 μg mitochondria. The assay was initiated, and the activity was measured for 2 to 3 min to ensure a stable baseline. Decylubiquinone (DQ, 50 μM) was added to each cuvette to start the reaction in the spectrophotometer (Shimadzu 2400UV) and the reaction rate was followed for 6 to 9 min. Rotenone (10 μM), a complex I inhibitor, was added to obtain the rotenone sensitive rates and the reaction was followed for a further 5 min. The rotenone sensitive rates were subtracted from the DQ rates, and the Beer–Lambert law (*A = ε x c x l*) was used to obtain the specific activity of the enzyme as shown in Equation 1. The specific activity was expressed as nmol/min/mg of protein. The molar extinction coefficient for NADH in the presence of DQ was set at 6810 M^−1^ cm^−1^.

(1) $$\text{Specific}\;\text{activity}\;\left(\text{nmol}/\min/\text{mg}\right)=\frac{\;\Delta \text{Absorbance}\;\text{per}\;\min\;}{\;\varepsilon x\;1000\;\left(1\;\text{mL}\;\text{cuvette}\;\text{volume}\right)\times \left(\text{mg}\;\text{protein}\;\text{added}\right)}$$

Complex II/III activity was measured using a method described by Spinazzi *et al.* (35). The reaction follows the reduction of cytochrome c using succinate as the electron donor, measured at 550 nm at 37 °C. The reaction mixture contained 100 mM phosphate, 1 mM succinate, 1 mM KCN, and 10 μg of isolated mitochondria made up to a final volume of 1 ml in each cuvette. The reaction was started by the addition of 50 μM oxidized cytochrome c, and the resulting increase in absorbance was measured for 6 to 9 min. Antimycin A (1 μM) was added to inhibit the reaction, and the antimycin A sensitive rates were followed for 5 min. The specific activity of the protein was expressed as nmol/min/mg of protein. The specific activity was calculated according to the Beer–Lambert law, as previously described in Equation 1, using a molar extinction coefficient for reduced cytochrome c was taken as 18,500 M^−1^ cm^−1^.

Complex IV–specific activity was measured according to the method published by Spinazzi *et al.* (35). In a 1 ml cuvette, 10 μg of isolated mitochondrial protein was added to a buffer containing 100 mM potassium phosphate pH 7. Baseline was read for 2 min before the addition of 50 μl 1 mM reduced cytochrome c. The reaction was followed for 5 min. The complex IV reaction adhered to zero order kinetics, so the calculation of the specific activity differed from other ETC complexes. The absorbance was taken at 0, 1, 2, and 3 min after addition of reduced cytochrome c. The absorbance at time 0 was taken as t = 0, and individual values for each time point were calculated using Equation 2:

(2) $$K_{1}=\left(t_{0}\;/t_{1}\right)/1$$

Rate constants were calculated for each time point, changing the denominator accordingly (1, 2, 3) and dividing by t_1_, t_2,_ t_3_. The average of these rates was taken as the rate of complex IV activity in min/mg.

Complex V assay is based on the method of J.W. Soper and P.L. Pederson, adapted in the laboratory of John B. Clark, 1979 (36). This method evaluates mitochondrial ATP synthase (complex V) activity by measuring the reverse reaction, that is, its ATPase activity. The action of ATP synthase is completely reversible and proportional to its ATPase activity. The reaction mixture contained 50 mM Tris buffer, 6 mM MgCl_2_, 100 mM KCl, 2 mM KCN, 1 mM rotenone, 5 mM phosphoenolpyruvate, 0.3 mM NADH, 6 mM ATP, 500 units/ml LDH, and 500 unit/ml pyruvate kinase in a 1 ml cuvette. The reaction was performed at 30 °C. Baseline activity was measured for 3 to 5 min, before the addition of 10 μg of mitochondrial protein. The subsequent rate was measured for 10 min. The oligomycin sensitive rate was determined by addition of 5 μM oligomycin, followed by measurement for a further 5 to 7 min. The insensitive rate was subtracted from the initial rate, and this was taken as the rate of ATP synthase activity. The specific activity of ATP synthase was calculated using Equation 1, taking the molar extinction coefficient of NADH as 6220 M^−1^ cm^−1^.

### Immunoblotting

Ten micrograms mitochondrial protein was loaded on to polyacrylamide gels, separated by SDS-PAGE and resolved proteins were transferred to polyvinylidene difluoride membranes using semidry transfer method. Membranes were blocked using 3% (w/v) BSA in PBS-Tween (0.1% Tween, (v/v)) for 1 h and incubated with primary antibody diluted in blocking buffer overnight. Primary antibody incubations were followed by incubation with the appropriate secondary antibody for 1 h at room temperature. Membranes were read on the Odyssey Infrared Imaging System (Li-COR), using Li-COR Application v3 software (www.licor.com) and images were adjusted using Image Studio Lite v4 software (www.licor.com).

Primary antibodies used were total OXPHOS Cocktail (Abcam, ab110413) and VDAC (Abcam, ab15895). Secondary antibodies used were goat anti-mouse IRDye 680RD (Li-COR, 925-68070) and goat anti-rabbit IRDye 800CW (Li-COR, 925-32211).

### RNA analysis by quantitative RT-PCR

Cells were seeded at 5 × 10^5^ cells/ml, allowed to adhere, and stimulated as indicated. Total RNA was extracted using the High Pure RNA Isolation Kit (Roche) and reversed transcribed with random hexamers (IDT) using Moloney murine leukemia virus reverse transcriptase (Promega) according to the manufacturers’ instructions. The resulting complementary DNA was analyzed by quantitative RT-PCR using the PowerUp SYBR Green Master Mix (Applied Biosystems) and gene-specific primer pairs (sequences are listed in Table S1). Relative mRNA expression was calculated by the comparative C_T_ method, normalizing the gene of interest to the housekeeping gene β-actin and comparing it to an untreated WT sample.

### Enzyme-linked immunosorbent assay

Quantification of secreted IL-10 from cell supernatants was by ELISA (R&D, DuoSet ELISA kits) following the manufacturers' instructions.

### Statistical analysis

Wilcoxon signed rank-test is a paired, nonparametric test and was chosen to compare pairs of biological replicates. Kruskal–Wallis test is a nonparametric test and was chosen to compare three or more groups of sample data. Statistics were calculated and graphs prepared with Excel 2016 (www.microsoft.com) and GraphPad Prism 9 (www.graphpad.com). Data are mean ± SEM; n.s. not significant, ∗*p* < 0.05, ∗∗*p* < 0.01, and ∗∗∗*p* < 0.001. Specific statistical tests are described in Figure legends.

## Data availability

All data are contained within the manuscript and in the Supporting information.

## Supporting information

This article contains Supporting information.

## Conflict of interest

The authors declare that they have no conflicts of interest with the contents of this article.
