# Supplementary material for: SARM1 regulates NAD+-linked metabolism and select immune genes in macrophages
Source: J Biol Chem. 2024 Jan 3;300(2):105620. doi: 10.1016/j.jbc.2023.105620 (PMC10847163; doi:10.1016/j.jbc.2023.105620)
Supplement: Supplemental Figs. S1–S5 [file mmc1.pdf]

Supporting Information

SARM1 regulates NAD$^+$-linked metabolism and select immune genes in macrophages

Katharine A. Shanahan[1], Gavin M. Davis[1], Ciara G. Doran[1], Ryoichi Sugisawa[1], Gavin P. Davey[1] and Andrew G. Bowie[1, 2, *]

[1]School of Biochemistry and Immunology, Trinity Biomedical Sciences Institute, Trinity College Dublin, Dublin 2, Ireland.

[2]Lead contact

*Correspondence: agbowie@tcd.ie

A

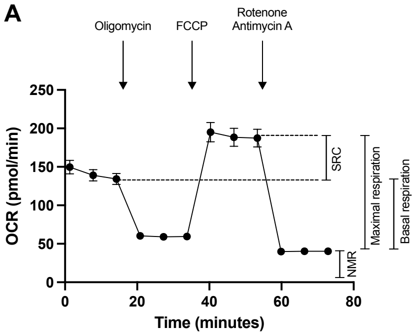

Parameter	Equation
Basal respiration	(Rate before first injection) - (NMR)
Maximal respiration	(Maximum rate after FCCP injection) - (NMR)
ATP-linked respiration	(Rate before oligomycin injection) - (rate after oligomycin injection)
Spare respiratory capacity (SRC)	(Maximal respiration) - (basal respiration)

B

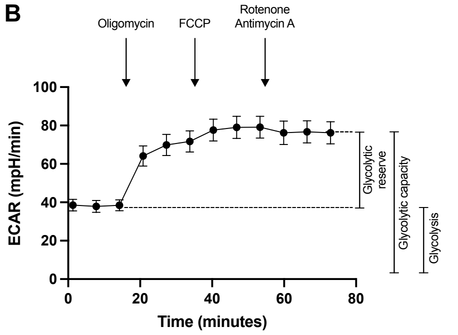

Parameter	Equation
Basal glycolysis	Rate before first injection
Glycolytic capacity	Maximum rate after oligomycin injection
Glycolytic reserve	(Glycolytic capacity) - (basal glycolysis)

Figure S1. Calculations for OCR and ECAR parameters from Seahorse traces.

(A) MitoStress test was selected to measure parameters of mitochondrial function by measuring oxygen consumption rate (OCR). OCR is represented in pmoles per minute (OCR (pmol/min)). OCR is modulated by sequentially injecting the mitochondrial inhibitors oligomycin, FCCP, rotenone and antimycin A. Arrows represent times of injection. OCR readings are taken at 6 minute intervals and 3 readings are recorded following each injection. Oligomycin, an inhibitor of ATP synthase, reduces electron flow through the ETC, decreasing OCR. FCCP uncouples the proton gradient from electron transport, allowing uninhibited electron flow through the ETC and therefore OCR reaches its maximum. Rotenone and antimycin A inhibit mitochondrial respiration, which reflects non-mitochondrial respiration (NMR). The parameters that can be determined are summarised in the table. (B) During glycolysis, the conversion of glucose to pyruvate and then lactate results in extrusion of protons into the extracellular medium, resulting in acidification. Changes in extracellular acidification, or the extracellular acidification rate (ECAR), are indicative of aerobic glycolysis. ECAR is measured alongside OCR during MitoStress test. Arrows represent times of injection. ECAR readings are taken at 6 minute intervals and 3 readings are recorded following each injection. Following inhibition of ATP synthase by oligomycin, ATP production switches to glycolysis, resulting in an increase in ECAR. The parameters that can be determined are summarised the table.

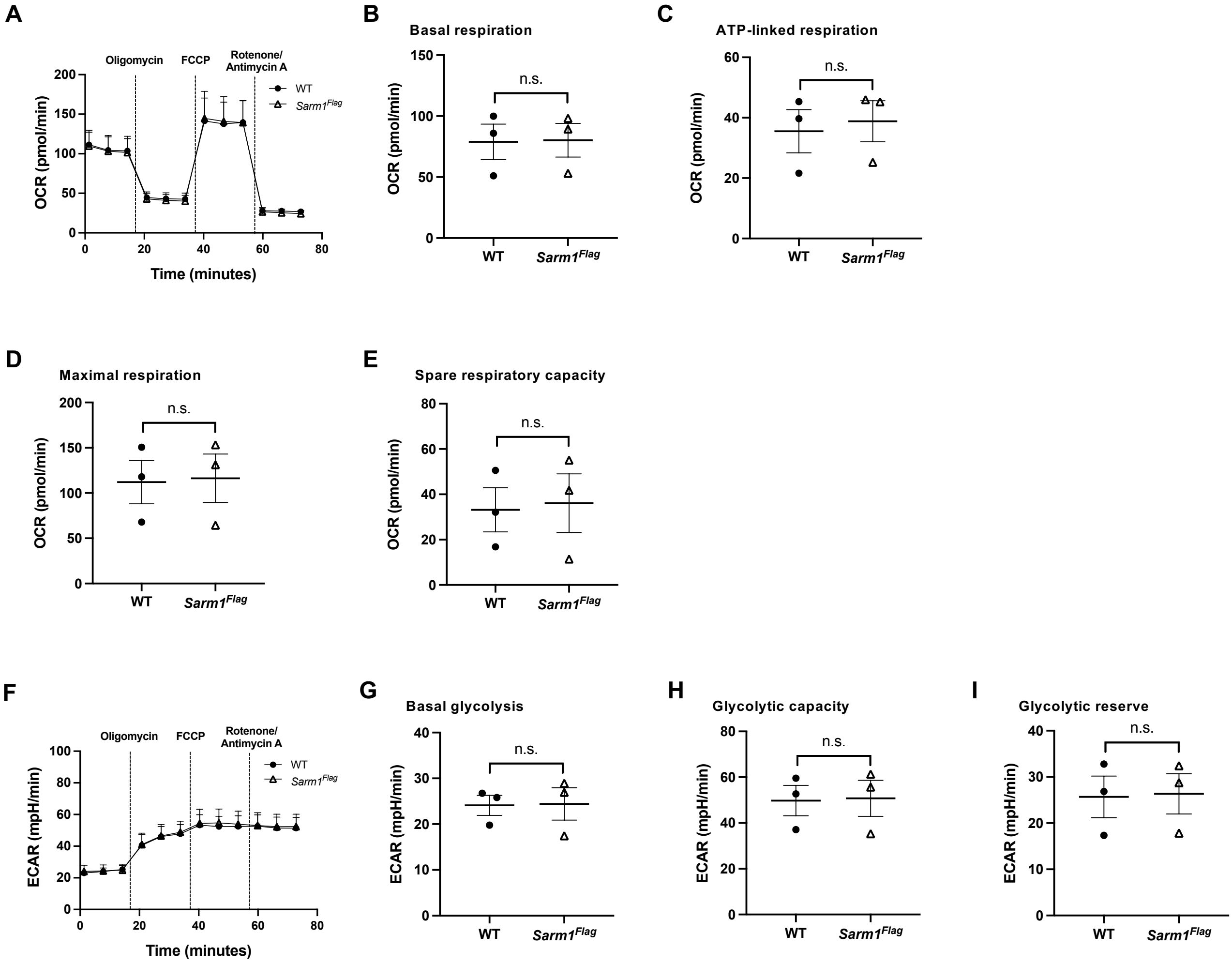

Figure S2. BMDM from *Sarm1^{Flag}* mice display similar oxygen consumption and glycolysis compared to WT macrophages.

Real time changes in OCR and ECAR in unstimulated WT and *Sarm1^{Flag}* BMDM were measured by Seahorse XF analysis. Representative OCR (A) and ECAR (F) traces of three independent experiments and each experiment was performed with six technical replicates. Dotted lines indicate injection times of mitochondrial inhibitors, oligomycin (1 μM), FCCP (1 μM), rotenone (1 μM) and antimycin A (2 μM). Basal respiration (B), maximal respiration (C), ATP-linked respiration (D), spare respiratory capacity (E), basal glycolysis (G), glycolytic capacity (H), and glycolytic reserve (I) were calculated and displayed as scattered dot plots. (B – E, G – I) Data are mean ± SEM from three independent experiments. Significance tested using two-tailed Wilcoxon matched-pairs signed rank test; n.s., no significant difference.

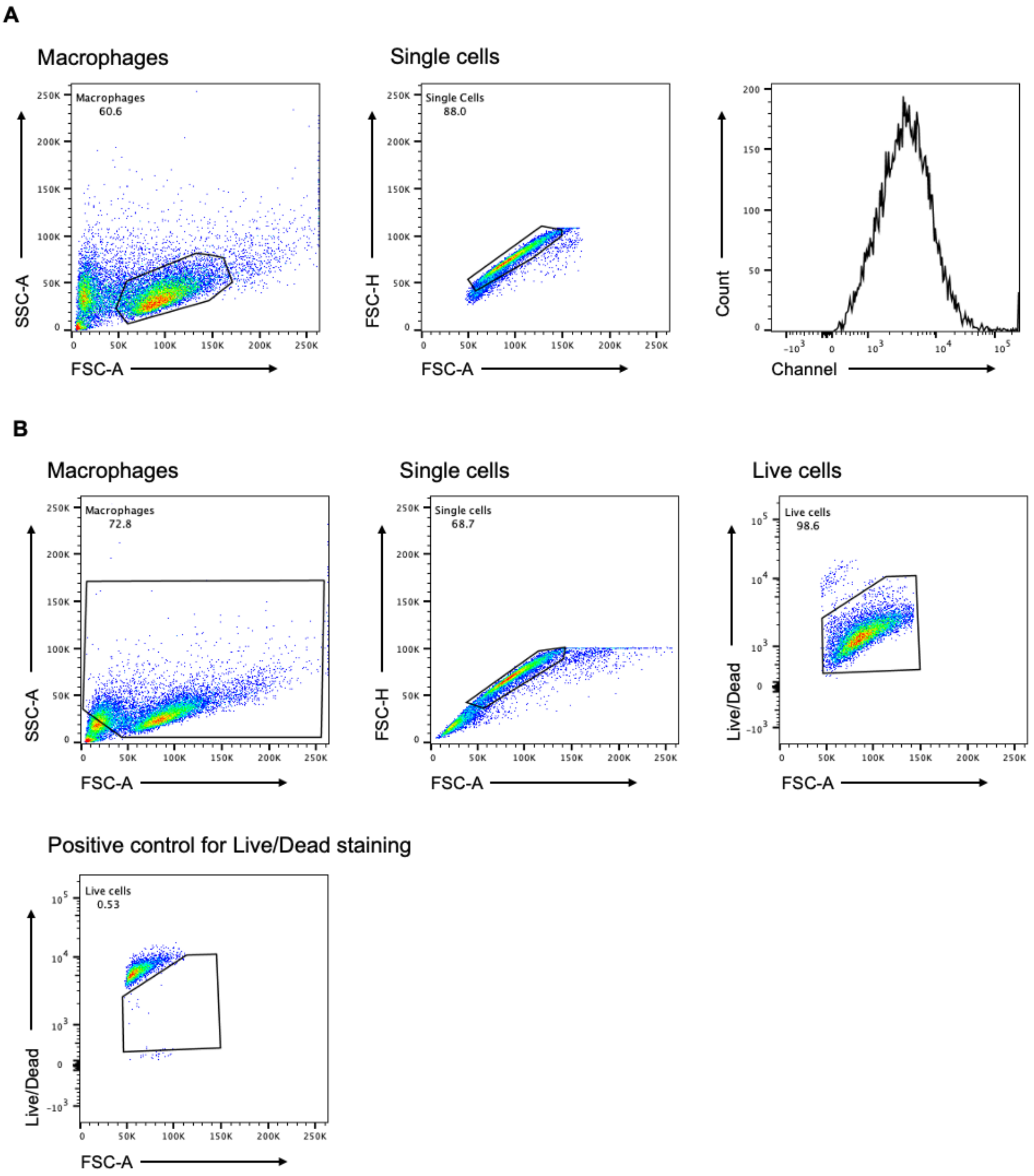

Figure S3. Gating strategy for unstimulated and treated macrophages.

(A) Live macrophage population was determined by side scatter area (SSC-A) and forward scatter area (FSC-A). Single cell population was determined by forward scatter height (FSC-H) and forward scatter area (FSC-A). (B) Total macrophage population was determined by SCC-A and FSC-A. Single cell population was determined by FSC-H and FSC-A. Live cells were selected by Zombie Aqua^{TM} staining. 100% ethanol was used as a positive control for Live/Dead staining.

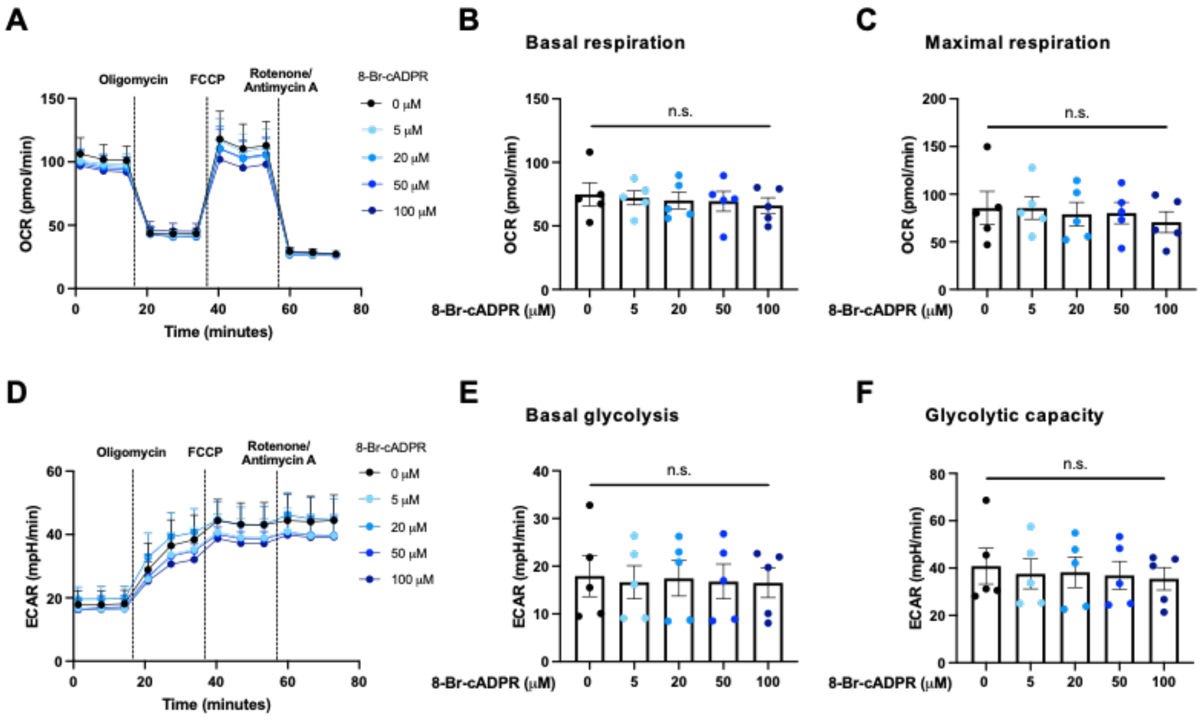

Figure S4. Pharmacological blockade of cADPR signalling does not affect maximal respiration or glycolysis in mouse macrophages.

(A – F) WT BMDM were treated with indicated concentrations of 8-Br-cADPR for 18 hours and real-time changes in OCR and ECAR were measured by Seahorse XF analysis. Representative OCR (A) and ECAR (D) trace of five independent experiments and each experiment was performed with six technical replicates. Dotted lines indicate injection times of mitochondrial inhibitors, oligomycin (1 μM), FCCP (1 μM), rotenone (1 μM) and antimycin A (2 μM). Basal respiration (B), maximal respiration (C), basal glycolysis (E) and glycolytic capacity (F) were calculated and displayed as bar charts. Data are mean ± SEM from five independent experiments. Significance tested using Kruskal-Wallis test; n.s., no significant difference.

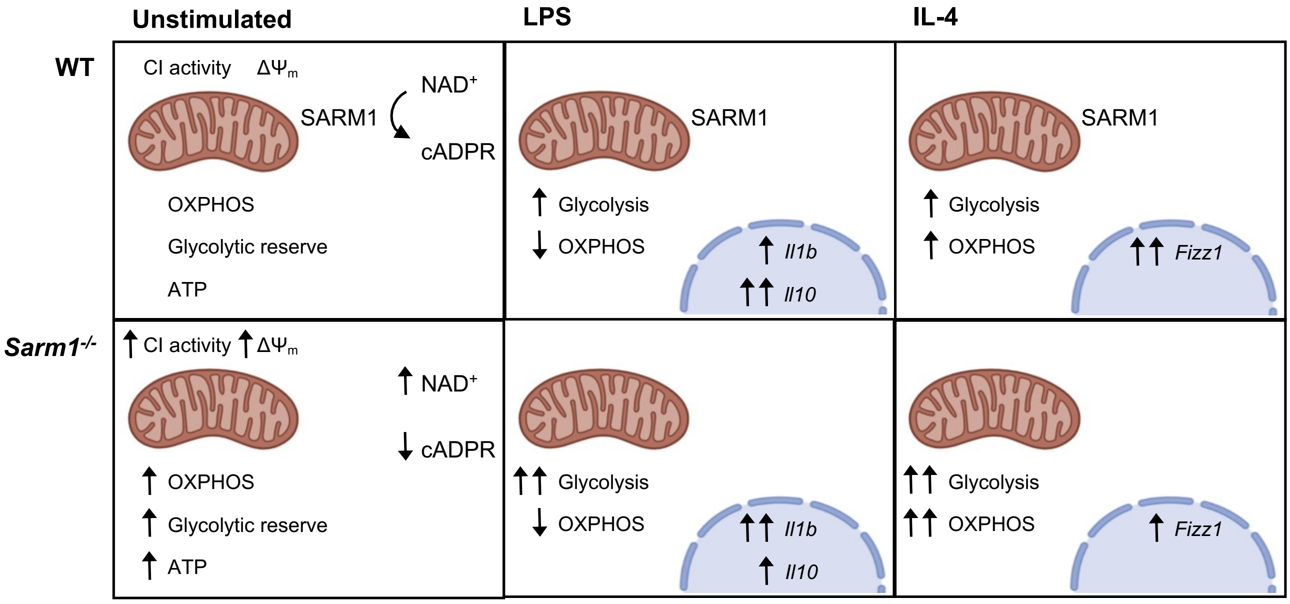

Figure S5. SARM1 modulation of cellular NAD$^+$ influences macrophage metabolism and select immune gene transcription.

In resting mouse macrophages, SARM1 cleaves NAD$^+$ to generate cADPR. SARM1 restricts the reserve capacity of oxidative phosphorylation (OXPHOS) and glycolysis and can limit complex I (CI) activity, $\Delta\Psi_m$ and cellular ATP concentrations. In pro-inflammatory macrophages, SARM1 limits upregulation of glycolysis, restricts expression of *Il1b* and enhances expression of *Il10*. In anti-inflammatory macrophages lacking SARM1, oxidative phosphorylation and glycolysis are increased and *Fizz1* expression is reduced.
