# Supplementary material for: SARM1 regulates NAD+-linked metabolism and select immune genes in macrophages
Source: J Biol Chem. 2024 Jan 3;300(2):105620. doi: 10.1016/j.jbc.2023.105620 (PMC10847163; doi:10.1016/j.jbc.2023.105620)
Supplement: Supplemental Table S1 [file mmc2.docx]

| *mt-ND1* | Fwd: TATCTCAACCCTAGCAGAAA |
| --- | --- |
|  | Rev: TAACGCGAATGGGCCGGCTG |
| *mt-CO1* | Fwd: GCCCCAGATATAGCATTCCC |
|  | Rev: GTTCATCCTGTTCCTGCTCC |
| *Pcam1* | Fwd: ATGGAAAGCCTGCCATCATG |
|  | Rev: TCCTTGTTGTTCAGCATCAC |
| *Il1b* | Fwd: GTGAAATGCCACCTTTTGACAGTGATGAG |
|  | Rev: CACCTTGGTCTTGGAGCTTAT |
| *Il6* | Fwd: AAGAGTTGTGCAATGGCAATTCTG |
|  | Rev: ATAGGCAAATTTCCTGATTATATCCAGT |
| *Il10* | Fwd: AGGCGCTGTCATCGATTT |
|  | Rev: CACCTTGGTCTTGGAGCTTAT |
| *Arg1* | Fwd: CTCCAAGCCAAAGTCCTTAGAG |
|  | Rev: AGGAGCTGTCATTAGGGACATC |
| *Fizz1* | Fwd: CAAGGAACTTCTTGCCAATCCAG |
|  | Rev: CCAAGATCCACAGGCAAAGCCA |
| *Mgl2* | Fwd: AACAGCTGGAGACAGACT |
|  | Rev: CAGACTCAGAGAACCAATAGC |
| *Ym1* | Fwd: CAGGTCTGGCAATTCTTCTGAA |
|  | Rev: GTCTTGCTCATGTGTGTAAGTGA |
| *Actb* | Fwd: TCCAGCCTTCTTCTTGGT |
|  | Rev: GCACTGTGTTGGCATAGAGGT |

**Table S1. Primers used for quantitative RT-PCR**
